# Mobile clinics in conflict-affected communities of North West and South West regions of Cameroon: an alternative option for differentiated delivery service for internally displaced persons during COVID-19

**DOI:** 10.1186/s13031-021-00427-9

**Published:** 2021-12-14

**Authors:** Lundi-Anne Omam, Elizabeth Jarman, Wilfred Ekokobe, Agbor Evon, Esther Njomo Omam

**Affiliations:** 1 Department of Public Health and Primary Care, University of Cambridge, Cambridgeshire, UK; 2 Health Department, Reach Out Cameroon, Buea, Cameroon

**Keywords:** Mobile clinics, HIV, Differentiated service delivery, COVID-19, Conflict-affected

## Abstract

**Introduction:**

The guidelines for differentiated service delivery (DSD) for HIV treatment became operational in Cameroon in 2017 with the Test and Treat national strategy elaborating services that can be decentralized and task shifted at community level, but with little to no guidelines for DSD in fragile and conflict-affected settings. Since 2016, more than 680,000 Cameroonians have been internally displaced due to the conflict in the North West and South West regions (NWSW). This conflict has impacted on the health system with numerous attacks on health facilities and staff, reducing access to health care for internally displaced persons. The outbreak of COVID-19 further reduced humanitarian responses for fear of spreading COVID-19. Mobile clinics were utilized as a model of care in piloting DSD for HIV in conflict-affected settings within the COVID-19 context.

**Methods:**

The HIV DSD framework was used to evaluate a project that used mobile clinics in 05 divisions across the NWSW to provide primary health care to internally displaced persons in hard-to-reach areas. These mobile clinics were operated in the COVID-19 context and integrated HIV services in the benefit package. The mobile clinics mainstreamed HIV and COVID-19 sensitization during community mobilization, HIV consultations, HIV testing and referrals, and in some cases antiretroviral (ARV) dispensation. The project ran from March to October 2020. The results from the evaluation of this model of HIV care delivery were analysed in 06 of 08 mobile clinics.

**Results:**

In 07 months, a total of 14,623 persons living in conflict-affected settings were sensitized on HIV, 1979 received HIV testing from which 122 were positive and 33 placed on ARVs. 28 loss-to-follow up people living with HIV were relinked to treatment and 209 consultations for persons living with HIV were conducted. Despite the good collaboration at regional and field level, there was distrust by ARV centers for humanitarian organizations.

**Conclusion:**

Mobile clinics are a model of care which could be leveraged in fragile and conflict-affected settings as an alternative model of care for HIV DSD to ensure continuum of HIV care and treatment. However this should be integrated within the benefit package of primary health care services offered by mobile clinics.

## Introduction

The availability of antiretroviral therapy (ART) for treatment of people living with HIV (PLHIV) has increased in resource limited settings. This has led to the need to develop and implement other treatment delivery models known as differentiated models of service delivery in areas of high prevalence of HIV [1]. Differentiated service delivery (DSD) refers to a model of care, made to effectively deliver patient-centred healthcare packages [2]. Four DSD models were described which focused on decentralization of delivery of ART and stability of ART clients [3]. This care plan was built based on the DSD framework defined by who provides care, where care is provided, what care is provided and when activities happen. The DSD was designed to improve retention in treatment within HIV programs in low-middle-income countries and has been globally adopted in national guidelines [2–4]. In Central and West Africa, Cameroon ranks second with respect to HIV burden [5]. Several efforts were made by the government of Cameroon to reduce the financial burden and geographical and social barriers of HIV [6]. Despite these efforts in 2016, only one-third of 585,276 people living with HIV (PLHIV) were retained on ARTs [7]. The government of Cameroon thus adopted the DSD model for HIV treatment which became operational in 2017, with testing and treatment services being decentralized and task shifted at community level [7]. Examples of strategies instituted to improve the care and ART treatment to PLHIV included (1) creating more HIV care units in the country to routinely follow-up PLHIV (2) devolving new HIV related tasks within primary health care facilities to trained non-medical staff and (3) introducing the community dispensation of ART through community-based organizations (CBOs) [8].These strategies unfortunately have no guidelines for DSD adaptation to the humanitarian context of conflict-affected settings of the North West and South West (NWSW) Regions of Cameroon.

For the past 4 years the english-speaking regions (NWSW) of Cameroon have been conflict-affected, with an estimated 680,000 internally displaced persons (IDPs) [9]. This crisis has had a major impact on the health sectors in these regions with an estimated 34% of health facilities in both regions being non-functional or partially functional with limited access to healthcare [10]. Following the outbreak of the COVID-19 pandemic, the immediate concern was the effect this pandemic will have on fragile health systems in Africa which were not ready for it [11]. With the outbreak of COVID-19 in Cameroon in March 2020 [12], it seemed obvious the impact would be devastating on health systems, especially those in conflict-affected zones.

In the midst of these two crises in NWSW Cameroon, mobile clinics were utilized as a model of care in piloting DSD for HIV in complex humanitarian settings. In this paper, we review and evaluate the mobile clinics model to identify best practices in piloting the implementation of integrated HIV DSD in fragile and conflict-affected settings and make recommendations for optimizing mobile clinics operations in fragile and conflict-affected settings.

## Methods

Across the North West and South West regions of Cameroon, displacement figures rose to 450,000–680,000 IDP population who sought refuge in the forests and farmlands, rural, urban and peri urban areas in the North West, South West Regions and in neighbouring Nigeria. This necessitated the use of delivery models like mobile clinics able to bring health care close to affected communities. The implementation of mobile clinics in the NWSW regions of Cameroon is a strategy adopted by the Health Cluster response plan in November 2019 to provide primary health care (PHC) to IDPs in hard-to-reach communities in conflict settings [13].

With funding from the World Health Organisation, a project entitled "Emergency Health response to limit morbidity and mortality among IDPs in the North West and South West" was implemented to provide PHC to conflict-affected communities using mobile clinics. Before the implementation of this intervention (mobile clinics), health service delivery in communities with non-functional health facilities was largely done by community health workers and community nurses trained and engaged by humanitarian organisations. Mobile clinics as an approach to provide PHC in the NWSW had been used before in Ekondotiti [14], but HIV services were not integrated in the package of care. Also, with the COVID-19 pandemic, adaptations had to be made to allow for essential services to be delivered in the context of a pandemic.

The targeted localities were in Fako, Kupe-Muanenguba, Manyu, Momo and Ngoketunjia divisions, with communities highly hit by the conflict. To ensure continuum of HIV care for IDPs in the COVID-19 context, HIV care and treatment services were integrated in the mobile clinics benefit package. HIV consultation, HIV testing, counselling with referrals, search of loss-to-follow cases and in some cases ARV administrations were the key HIV services offered by the mobile clinics. All daily data from the mobile clinics were captured on HIV log registers and entered into excel sheets for analysis and reporting. These report were transferred electronically by email to the project manager on a weekly base who compiled all reports received in an excel sheet. All patients had a patient code, and management of patient data was limited to the team leader (mobile clinic doctors) and the project management team at the head office.

To identify DSD relevant practices using mobile clinics in conflict settings, we reviewed the reports and database from mobile clinics that were operated in 5 divisions. The following eligibility criteria for inclusion were used: (1) mobile clinics delivered PHC in hard-to-reach conflict settings, (2) mobile clinics integrated HIV services as part of the benefit package (3) mobile clinics with reported data on HIV service delivery. Nine months reports and database (March to October 2020) were reviewed from December 2020 to February 2021 and 06 of the 08 mobile clinics were selected for inclusion in this study (two mobile clinics were excluded due to poor reporting on HIV service delivery). We adapted a framework (Table1) to allow for a detailed program description using the 04 building blocks of DSD [12] to suit our context and model of health care, and we clearly looked for examples of DSD due to the potential of this strategy to ensure continuity of HIV services at community level especially in hard-to-reach conflict-affected areas.

**Table 1:** Framework for the evaluation of 6 mobile clinics in conflict settings

DSD building blocks	Questions
Service providers	Who were the service providers in the mobile clinics?
	What role did they have within the mobile clinics?
Location of services	How were target locations originally identified (i.e. criteria)?
	What were the target populations and locations of mobile clinics?
	How many communities were covered per mobile clinic?
	What were the facilitators and barriers to reaching intended locations and populations?
	What were the reasons for changing target locations/populations during the project?
Frequency of services	What was the average rotation time per community?
	Did covid-19 impact the frequency and rotation of mobile clinics?
	How did actual frequency compare with planned frequency?
	What were the facilitators and barriers to achieving planned frequency of services?
Benefit package (Package of services	What PHC services did the mobile clinics offer?
	What HIV services did the mobile clinics offer? (Consultations, testing, ARV refills, contact tracing, LTFU, counselling, referrals, viral load monitoring?)
	How many people were screened for HIV, how many PLHIV were linked or initiated on ARV? How many loss to follow up cases were relinked to treatment?
	What COVID-19 infection prevention and control measures were implemented to ensure continuity of health service delivery?
	How did COVID-19 infection prevention and control measures affect the composition or delivery of the services in the benefit package?

### Ethical considerations

This evaluation solely made use of secondary data available in field reports and database, and so, there was no need to seek ethical approval.

## Results

### Service providers

Each team of service providers was made up of 04 medical personnel; a medical doctor and three nurses (Table 2). During community activities, 01 nurse was in charge of triage and their role was to fill the patient card with patient’s information. Vital signs and anthropometric measurements including blood pressure, temperature, pulse, respiratory rate, weight and mid-upper arm circumference of children 6 months to 5 years. The doctor conducted consultations and emergency management and completed the outpatient register. A second nurse conducted laboratory investigations including malaria rapid diagnosis test, venereal disease research laboratory test, beta human chorionic gonadotropin, HIV, urinalysis, hemoglobin level and blood sugar. Results were recorded on the patient card and the laboratory register. A third nurse managed the pharmacy and dispensed prescribed medications, dressed wounds and conducted vaccination.

**Table 2:** General overview of included mobile clinics using the framework

Domains	Fako	Kupe-Muanenguba 1	Kupe-Muanenguba 2	Manyu	Ngo-ketunja 1	Ngo-ketunja 2
Service providers	01 medical doctor for consultations	02 medical doctor for consultations		01 medical doctor for consultations	02 medical doctor for consultations	
	03 nurses for laboratory, triage and pharmacy	06 nurses for laboratory, triage and pharmacy		03 nurses for laboratory, triage and pharmacy	06 nurses for laboratory, triage and pharmacy	
	Driver for team transportation, crowd control	Driver for team transportation of both teams, crowd control for one team		Driver for team transportation, crowd control	Driver for team transportation of both teams, crowd control for one team	
	Safety officer for community mobilisation and access	Safety officer for community mobilisation and access for both team		Safety officer for community mobilisation and access	safety officer for community mobilisation and access for both team	
Location of services	Targeted IDP and host population in rural, peri-urban	Targeted IDP and host populations in rural communities	Targeted IDP and host population in peri-urban communities	Targeted IDP and Host population in rural communities	Targeted IDP and host populations in peri-urban and urban	Targeted IDP and host populations in Urban communities
	15 communities	8 communities	8 communities	20 communities	Communities	5 communities
	47,935 IDP population*	10,009 IDP population*		25,003 IDP population*	5 communities	
					16,199 IDP population*	
Frequency of services	*Planned*: Weekly	*Planned*: Fortnightly	*Planned:* Fortnightly	*Planned:* Fortnightly	*Planned:* Weekly	*Planned:* Weekly
	*Actual:* Fortnightly—monthly	*Actual*: Monthly	*Actual:* Monthly	*Actual:* Monthly	*Actual*: Weekly—fortnightly	*Actual*: Weekly—fortnightly
Benefit pages	Consultations, testing, contact tracing, counselling, referrals,	Consultations, testing, contact tracing, counselling, referrals		Consultations, testing, contact tracing, counselling, referrals, ARV dispensation, ARV refills	Consultations, testing, contact tracing, counselling, referrals	

^*^Estimate data from the multisector needs assessment of 2019

### Location of services

Communities were originally targeted using data from the 2019 multi-sector needs assessments (MSNA). Districts and communities were chosen following two criteria; lack of functional health facilities, and access (safety). All age groups suffering from health problems benefited from the services with focus on children under 5 years, pregnant and lactating women, people living with disability and the elderly. Due to safety constraints in NWSW regions, community targeting was a huge challenge. Communities that could not be accessed on a planned date due to security concerns were rescheduled to be visited on a later date. Most communities in the 05 divisions of implementation benefited from the health services offered by the mobile clinics. A total of 20 communities were targeted in Manyu, 15 communities in Fako, 10 in Ngo-ketunjia and 16 in Kupe-Muanenguba Division (Table 2). Of these communities, 27 were IDP settlements and 34 were host communities.

Poor roads, bad communication network and insecurity from gunshots, kidnapping or forceful retention were the main barriers to reaching intended locations and populations. However, with the support of community facilitators and community leaders who mobilised the communities, the mobile clinics where able to access most targeted communities. Also, with the use of visibility materials like flags, large project stickers on the vehicle, and project jackets with logos, it was easy to identify the mobile clinic teams from a distance. Also, the use of a 4 × 4 Hilux facilitated transport to communities with very bad roads even during the rainy season. Although the aforementioned strategies were used to access most communities, some target locations could still not be accessed due to security constraints linked with “Ghost towns or lockdowns” and arm confrontations between belligerents.

### Frequency of services

The mobile team targeted hard-to-reach communities in selected divisions. This accounts for the fact that all teams didn’t have equal number of communities because some communities were inaccessible because of bad roads and/or insecurity. However, the teams had a work or rotation plan which permitted them to revisit targeted community at least once a month and at most four times a month in some communities. The team in Manyu covered more communities than the other teams and so had less frequent visits whereas Ngo-ketunjia covered fewer communities, with higher population, so conducted weekly visits.

### Benefit package

The objective of the mobile clinic was to provide PHC to communities that had difficulty accessing this. The following services were carried out in all mobile health clinics: general consultation, testing and treatment of communicable diseases, sensitization on health events especially with the outbreak of COVID-19, provider initiated counselling and testing for HIV, linking of HIV positive cases and lost to follow up (LTFU) cases to the nearest HIV treatment centres, refill of ARVs (Manyu division only), initiation of ARVs (Manyu division only), antenatal care, vaccination, family planning services and referral of patients to higher category facility.

#### Provider initiated HIV testing

A total number of 1979 (928 males, 1051 females) people were tested for HIV in 04 divisions (Manyu, Fako, Kupe-Muanenguba and Ngo-ketunjia) in NWSW (Table 3). Manyu had the highest number of persons tested 595 (30%) and Ngo-ketunjia had the lowest number of people tested 404 (20.4%). Out of the four divisions, 122/1979 (6%) tested positive and 1857/1979 (94%) tested negative. Ngo-ketunjia Division recorded the highest number of positive cases 67/404 (17%) and Fako division recording the least number of positive cases 9/453 (2%).

**Table 3:** Summary HIV achievements using mobile clinics in DSD

Divisions	Number of general consultations		Number of HIV consultations		Number sensitized		Number tested for HIV		Number tested positive	
	*M*	*F*	*M*	*F*	*M*	*F*	*M*	*F*	*M*	*F*
Fako										
Urban	743	1156	2	6	240	213	72	80	1	1
Peri Urban	560	693	1	3	200	229	45	53	1	1
Rural	570	807	1	4	347	455	106	97	2	3
**Fako total**	**1873**	**2656**	**4**	**13**	**787**	**897**	**223**	**230**	**4**	**5**
Kupe Muanenguba 1										
Urban	560	630	4	8	98	98	74	46	1	4
Peri Urban	435	517	2	9	56	94	68	35	0	2
Rural	893	862	6	20	146	172	107	94	3	3
**Kupe Muanenguba 1 Total**	**1888**	**2009**	**12**	**37**	**300**	**364**	**249**	**175**	**4**	**9**
Kupe Muanenguba 2										
Urban	190	335	2	4	210	229	12	17	1	0
Peri Urban	230	210	2	3	190	166	9	11	1	0
Rural	518	650	4	13	330	601	25	29	2	0
**Kupe Muanenguba 2 Total**	**938**	**1195**	**8**	**20**	**730**	**996**	**46**	**57**	**4**	**2**
Manyu										
Urban	980	885	1	12	600	710	72	84	0	5
Peri Urban	450	540	1	9	408	512	51	76	0	3
Rural	1229	1232	3	32	1110	1104	150	162	3	16
**Manyu Total**	**2659**	**2657**	**5**	**53**	**2118**	**2326**	**273**	**322**	**3**	**24**
Ngo Ketunjia 1										
Urban	330	403	4	8	463	421	35	29	3	8
Peri Urban	323	361	3	9	396	302	28	37	3	7
Rural	505	879	8	17	733	1390	53	130	7	16
**Ngo Ketunjia 1 Total**	**1158**	**1643**	**15**	**34**	**1592**	**2113**	**116**	**196**	**13**	**31**
Ngo Ketunjia 2										
Urban	323	506	0	2	210	306	4	13	1	3
Peri Urban	295	394	0	3	195	410	6	9	2	4
Rural	548	1062	0	2	451	828	11	49	5	8
**Ngo Ketunjia 2 Total**	**1166**	**1962**	**0**	**7**	**856**	**1544**	**21**	**71**	**8**	**15**
**Grand Total**	**9682**	**12,122**	**44**	**165**	**6383**	**8240**	**928**	**1051**	**36**	**86**

Divisions	Number tested HIV positive (new cases) and placed on ARV		Number of PLHIV already on treatment (old cases but tested)		Number of LTFU PLHIV seen		Number of LTFU relinked to treatment		Number of LTFU relink to treatment center but poor documentation	
	*M*	*F*	*M*	*F*	*M*	*F*	*M*	*F*	*M*	*F*
Fako										
Urban	1	0	1	2	1	2	0	0	0	0
Peri Urban	0	0	0	0	0	0	0	0	0	0
Rural	1	2	0	1	1	2	0	1	0	
**Fako total**	**2**	**2**	**1**	**3**	**2**	**4**	**0**	**1**	**0**	**0**
Kupe Muanenguba 1										
Urban	0	1	3	4	1	2	1	1	0	0
Peri Urban	0	0	2	2	0	2	0	1	0	1
Rural	1	1	2	5	2	3	0	2	1	1
**Kupe Muanenguba 1 Total**	**1**	**2**	**7**	**11**	**3**	**7**	**1**	**4**	**1**	**2**
Kupe Muanenguba 2										
Urban	1	0	1	1	1	3	1		0	0
Peri Urban	0	1	0	2	1	1	0	0	0	0
Rural	0	1	1	2	1	3	2	1	0	0
**Kupe Muanenguba 2 Total**	**1**	**2**	**2**	**5**	**3**	**7**	**3**	**1**	**0**	**0**
Manyu										
Urban	0	0	1	1	2	4	0	2	0	0
Peri Urban	1	1	0	1	0	3	0	3	0	0
Rural	0	2	1	3	0	9	1	2	0	0
**Manyu Total**	**1**	**3**	**2**	**5**	**2**	**16**	**1**	**7**	**0**	**0**
Ngo Ketunjia 1										
Urban	2	3	0	0	1	4	0	0	1	1
Peri Urban	2	1	0	1	2	4	1	2	0	1
Rural	2	8	1	2	3	10	1	0	0	1
**Ngo Ketunjia 1 Total**	**6**	**12**	**1**	**3**	**6**	**18**	**2**	**2**	**1**	**3**
Ngo Ketunjia 2										
Urban	0	0	0	1	0	4	1	1	0	1
Peri Urban	0	1	0	0	2	3	1	0	0	1
Rural	0	0	0	1	1	8	1	2	0	0
**Ngo Ketunjia 2 Total**	**0**	**1**	**0**	**2**	**3**	**15**	**3**	**3**	**0**	**2**
**Grand Total**	**11**	**22**	**14**	**30**	**19**	**59**	**10**	**18**	**2**	**11**

#### HIV consultations

Of the 26,782 expected general consultations, 21,804 was achieved from which 209 HIV consultations were conducted (44 males, 165 females). These included addressing medical problems of PLHIV, ensuring adequate supply of co-trimoxazole and treatment of opportunistic infections as well as initiation on ARVs (antiretroviral drugs). 33/122 persons newly tested HIV positive were placed on ARV and 44/122 who tested positive were already on treatment (Table 3). This was the 20th most popular consultation of the mobile clinics.

#### Sensitization

14,623 (6383 males, 8240 females) people were sensitized on HIV as part of the clinics, and 18,112 (7770 males, 10,342 females) were sensitized on COVID-19. Sensitization was done by the medical team before patient consultation with the doctor. This was the first activity the clinics started their daily activity with and repeated the lectures after every 2 h to ensure that all patients received the educative message irrespective of their time of arrival at the mobile clinic.

#### Linking

78 LTFU PLHIV were identified through the clinics. Of these, 28 were successfully linked to a treatment centre and reinitiate on ARVs, while 13 LTFU were linked to treatment but were poorly documented by the mobile clinics.

#### Changes to the mobile clinics design due to COVID-19

The mobile clinics had modified their targeting and crowd control strategy to reduce the number of persons who can showed up at the mobile clinic at any time. Community leaders, which were used to inform their communities to bring their facemasks to the mobile clinics. Hand wash stations were set up in front of the mobile clinics. In a bid to respect COVID-19 infection prevention and control measures during service delivery, patients sitting arrangement in the waiting areas were spaced out to respect a 2-m gap. This was however challenging in communities with no big community hall were sitting arrangements could not accommodate the 02 m gap. Under such circumstances, the mobile clinic team created a waiting area outside of the hall.

## Discussion

Treating every PLHIV is essential to achieve viral suppression for all by 2030 [15], yet many people in conflict-affected settings are still missing out on ARV treatment [16]. Various DSD models are being used to increase access to ARV in difficult to reach settings [3]. These include but are not limited to, home based ART dispensation, patient-led community ART distribution points, community ART groups, and mobile clinics [17–20].

Using mobile clinics as a model of care for DSD makes use of skilled personnel as seen in our 06 clinics (Table 2). This might be logistically challenging and costly to maintain in the long term [21]. But if HIV services are mainstreamed in the general benefit package of PHC offered by the mobile clinics, it may be cost effective within humanitarian settings. With the COVID-19 outbreak, the use of mobile clinics to ensure the continuum of essential services in conflict settings became even more useful. However, there is need to conduct cost-effectiveness studies on the use mobile clinics for HIV DSD in conflict settings post COVID-19.

Mobile clinics are commonly used in conflict-affected settings to provide PHC [21] and HIV services in rural hard-to-reach settings [20, 22], even though they have been reported to be expensive, logistical burdensome, ill-fitted to address chronic diseases, and may be limited in coverage to address acute illnesses. Infact, it is recommended that mobile clinics be used as “last resort” to reach populations who are cut off from health services [21, 23]. The mobile clinics in this project targeted peri-urban and rural communities affected by the conflict. The clinics rotated weekly to monthly between communities depending on the number of communities and the populations they had to cover in the division. This model might be suited for DSD as HIV services and treatment could be brought closer to communities at a frequent interval [3, 15].

A total of 6.2% of persons tested by mobile clinics were positive in these regions, which is above the national prevalence (4.3%) but almost similar to the regional prevalence (5.3% SW and 6.3% NW). This highlights the importance of having evidence based DSD for HIV specific for this settings. Through 06 mobile clinics in NWSW Cameroon, 33 out of 122 newly tested PLHIV living in conflict settings were started on ARV. Only 28 of the 78 LTFU cases identified were successfully linked to care with evidence of counter referral forms. The inability to successfully link all or many defaulters back to ARV treatment was a shortcoming of our mobile clinic model. In such settings with a fragile health system and limited access to health care, our mobile clinics were suppose to address this gap but failed to do so. This gap might have been due to the distance required for PLHIV to travel to receive ARV, coupled to the high levels of insecurity in these communities [24]. Cost of transportation may also have accounted for the non-successful linkage to care. For those who were successfully relinked to ARV, the mobile clinic (Mamfe) dispensed ARV to them in the forests and bushes where they were settled. The team in Mamfe successfully collaborated with the treatment centre to supply ARV for PLHIV they met in the bush and hard-to-reach communities. This level of collaboration and trust from the treatment centres to allow the mobile clinics deliver ARVs was lacking in the other divisions. Studies to ascertain why all PLHIV could not be successfully relinked to ARV need to be conducted in these settings.

Up to 44 PLHIV who knew their status were tested at the mobile clinics. During consultations, these patients hid their status but later on confessed knowing their status after laboratory confirmation. We speculate these PLHIV sort to consult for free and thus hid their status in the process. Previous studies have shown the impact financial barriers has on HIV programming [25].

To ensure complementary system strengthening approaches to HIV care and treatment are implemented, guidelines for ARV treatment and management for IDPs need to be developed by the National AIDS Control Committee. Similar guidelines for displaced persons have been developed in South Africa as mentioned by Hanson et al. [16].

This paper solely presented the experiences and results of providing differentiated service delivery for HIV using mobile clinic interventions and our results are liable to bias. Also, the framework used in Table 1 only allowed for a description of our program. A critical evaluation of the mobile clinics interventions and its outcomes in these conflict settings is required. Further, it was difficult to make a thorough appraisal on the performance of the achievements due to the lack of baseline and targets for the results presented in this paper.

## Conclusion

During the COVID-19 pandemic, mobile clinics offered an opportunity to deliver HIV DSD for IDPs in conflict-affected communities, though there is limited evidence on the efficiency and effectiveness of the model of care for HIV DSD in conflict settings. We recommend longitudinal or randomised controlled trials to be conducted on the use of mobile clinics in conflict settings as a model of care in HIV DSD. If mobile clinics are to be used in conflict settings for HIV DSD, this should be integrated within the package of PHC services offered in mobile clinics. Using mobile clinics as a stand-alone intervention for HIV DSD in conflict settings may be very costly. However, economic evaluation studies on the use of mobile clinics in for HIV DSD in conflict settings should be conducted.

## Data Availability

The datasets used during the current project are available from the corresponding author on reasonable request.

## Abbreviations

DSD: Differentiated service delivery
IDP: Internally displaced persons
LTFU: Lost to follow up
PLHIV: People living with HIV
ARV: Antiretroviral
PHC: Primary health care
NWSW: North West and South West regions
